# Development of Lentivirus-Based Reference Materials for Ebola Virus Nucleic Acid Amplification Technology-Based Assays

**DOI:** 10.1371/journal.pone.0142751

**Published:** 2015-11-12

**Authors:** Giada Mattiuzzo, James Ashall, Kathryn S. Doris, Kirsty MacLellan-Gibson, Carolyn Nicolson, Dianna E. Wilkinson, Ruth Harvey, Neil Almond, Robert Anderson, Stacey Efstathiou, Philip D. Minor, Mark Page

**Affiliations:** National Institute for Biological Standards and Controls-MHRA, South Mimms-Potters Bar, United Kingdom; Division of Clinical Research, UNITED STATES

## Abstract

The 2013-present Ebola virus outbreak in Western Africa has prompted the production of many diagnostic assays, mostly based on nucleic acid amplification technologies (NAT). The calibration and performance assessment of established assays and those under evaluation requires reference materials that can be used in parallel with the clinical sample to standardise or control for every step of the procedure, from extraction to the final qualitative/quantitative result. We have developed safe and stable Ebola virus RNA reference materials by encapsidating anti sense viral RNA into HIV-1-like particles. The lentiviral particles are replication-deficient and non-infectious due to the lack of HIV-1 genes and Envelope protein. Ebola virus genes were subcloned for encapsidation into two lentiviral preparations, one containing NP-VP35-GP and the other VP40 and L RNA. Each reference material was formulated as a high-titre standard for use as a calibrator for secondary or internal standards, and a 10,000-fold lower titre preparation to serve as an in-run control. The preparations have been freeze-dried to maximise stability. These HIV-Ebola virus RNA reference materials were suitable for use with in-house and commercial quantitative RT-PCR assays and with digital RT-PCR. The HIV-Ebola virus RNA reference materials are stable at up to 37°C for two weeks, allowing the shipment of the material worldwide at ambient temperature. These results support further evaluation of the HIV-Ebola virus RNA reference materials as part of an International collaborative study for the establishment of the 1^st^ International Standard for Ebola virus RNA.

## Introduction

The 2013-present Ebola virus outbreak in Western Africa has been the largest outbreak of Ebola virus with 27,872 laboratory confirmed cases, and 11,281 deaths as of 5th August 2015 [1]. The World Health Organisation (WHO) declared the Western Africa outbreak a Public Health Emergency of International Concern under the International Health regulations (2005). Nucleic acid amplification technology (NAT)-based diagnostic assays have been central to clinical management and control of this outbreak [2,3]. These assays have been applied to both diagnose and discriminate Ebola virus disease from other fevers, and to screen clinically asymptomatic Ebola virus disease patients prior to discharge from hospital [4]. Several in-house and commercial NAT-based assays have been developed (reviewed in [5]), however there are no globally available references to support the standardisation, control and assessment of these assays. Furthermore, the difficulties of undertaking assays in the field and the challenges in interpreting Ebola virus PCR results have been reported [6]. Both could result in false negative results. The availability of reference materials for the comparison of the sensitivity of different assays, for the validation of recently developed point-of care technologies and for the harmonisation of inter- and intra-laboratory results is therefore fundamental. This objective has been endorsed by the WHO Advisory group on Ebola virus disease response (David Wood, WHO, personal communication).

An ideal NAT reference material should be applied to the whole procedure including extraction. Current in-house and commercial assays utilise plasmids or *in vitro*-transcribed RNA as references for the assays [5,7,8]. Such reference materials do not control for the extraction procedure and may therefore contribute to false negative results. Wild type virus preparations have been used as controls [9,10], but these are limited to those laboratories which have access to bio-containment level 4 facilities. Inactivation of high titre virus stock by a single method does not assure safety, and most published procedures that produce viral antigen through a combination of methods [11], also result in disruption of viral RNA making its suitability as NAT standard uncertain [12]. To control for efficiency of the extraction procedure, and the presence of PCR inhibitors, clinical samples can be spiked with a bacteriophage such as MS2 [13–15] (and discussed at Technical Workshop on the Standardisation of Serological and PCR assays for the detection of Ebola virus NIBSC, UK, 5–6 March 2015-report in preparation); however this strategy involves the use of two standards, one for the Ebola virus RNA and one for the bacteriophage control, requiring either multiplexing with the risk of lowering amplification efficiency or doubling the number of reactions performed.

We have developed novel Ebola virus reference materials by encapsidating the Ebola virus anti-sense RNA within human immunodeficiency virus type 1 (HIV-1)-like particles. The final product, HIV-EBOV RNA, is a safe, non-infectious, non-replicating, freeze-dried preparation which could act as a reference material for the entire procedure from extraction through to amplification. Four reference materials have been produced and validated at NIBSC: two high-titre materials which can be used as calibrators for secondary standards, and two low-titre materials to serve as in-run controls.

## Materials and Methods

### Construction of the lentiviral vectors containing Ebola virus genes

**Fig 1:**
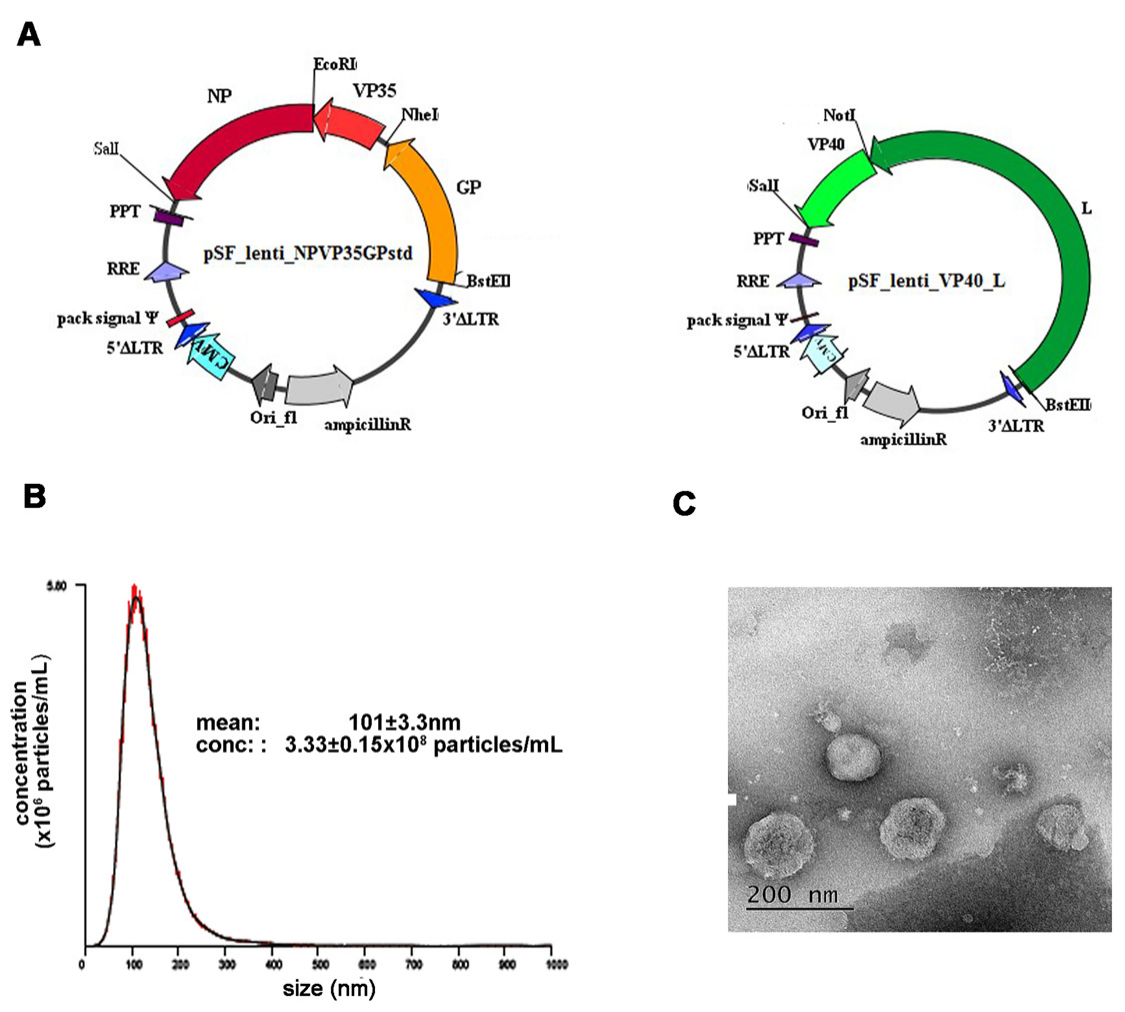
Generation of lentiviral particles containing Ebola virus RNA. A) Ebola virus genes nucleoprotein (NP, dark red), viral protein 35 (VP35, red), glycoprotein (GP, orange), viral protein 40 (VP40, light green) and the polymerase encoding gene L (dark green) were sequentially cloned into lentiviral vector pSF_lenti between the restriction sites SalI and BstEII. The main elements of the lentiviral vector used for the production of the viral RNA and incorporation within HIV-like particle are indicated. B) Particle size distribution of the 1:100 dilution in PBS of stock preparation of lentiviral particles containing Ebola virus RNA for *np-vp35-gp* genes. The graph represents the mean of 5 acquisitions (black line) ± standard deviation (red line). C) A representative image of the same preparation diluted 1:10 in PBS analysed by negative staining transmission electron microscopy. The average particle size was 116.02 nm (average of 9 fields).

Ebola virus nucleotide sequences were derived from *Zaire ebolavirus* isolate H.sapiens-wt/GIN/2014/Makona-Kissidougou-C15, GenBank accession number KJ660346.2 [16]. Sequences were modified to contain random stop codons and enzymatic restriction sites at the 5´ and 3´ end as illustrated in Fig 1A. Each gene was synthesised by GeneWiz Inc. and cloned into pUC57-Kan plasmid. Lentiviral vector pSF-lenti-PGK-FLuc is a customised version of the pSF-lenti (OG269, Oxford Genetics) in which the Cytomegalovirus (CMV) major immediate early promoter (MIEP) upstream of the multi cloning site has been removed and the Puromycin resistance gene has been substituted with the reporter gene Firefly luciferase. Each Ebola virus gene was sequentially subcloned from pUC57-Kan plasmid into the pSF-lenti-PGK-Fluc using standard molecular techniques. Final plasmid sequences pSF-lenti-NP-VP35-GP and pSF-lenti-VP40-L were confirmed by sequencing using Nextera XT library preparation kit (Illumina), following the manufacturer’s instructions, and sequenced on a MiSeq 2 × 251 paired-end v2 Flow Cell (Illumina). Results were analysed using Geneious R7 version 7.1.7(Biomatters) and deposited in GenBank (accession number KT186367 and KT186368, respectively).

### Generation of the HIV-EBOV RNA preparations

Lentiviral particles were generated by transfection of 5x10^6^ HEK 293T-17 (ATCC CRL-11268) cells in a 10cm dish with a mixture of 18 μL of FuGene-6 transfection reagent (Promega) and 1.5 μg of p8.9 [17] and 2 μg of pSF-lenti-NP-VP35-GP or pSF-lenti-VP40-L in 200 μL of Optimem (Gibco). After 20 minutes at room temperature, the mixture was added drop-wise to the cells in 8 mL of Dulbecco modified essential media (DMEM, Gibco) supplemented with 10% foetal calf serum (PAA) and the cells were cultivated at 37°C with 5% CO_2_. Supernatant was harvested at 48 and 72 hours post transfection and filtered using a 0.45 μm filter (Millipore). Supernatants collected at the different time points were pooled and treated for 1 hour at 37°C with 500 U/mL of DNase I (Life Technologies). Particles were purified by ultracentrifugation for 2 hours at 23,000rpm at 4°C on a 20% sucrose cushion in 50mM sodium phosphate buffer using a SW28 rotor in a Beckman Optima LE-80K Ultracentrifuge. Pellets were resuspended in an equivalent volume of universal buffer, composed of 10mM Tris-HCl pH 7.4, 0.5% Human Serum Albumin (Bio Products Laboratory Limited), 0.1% D-(+)-trehalose dehydrate (Sigma). High titre preparations were obtained by dilution of the purified particles 1:10 in universal buffer; low titre preparations were produced by further 1:10,000 dilution of the high titre material in universal buffer. All preparations were kept refrigerated overnight and were freeze/dried within 24 hours.

### Freeze-drying procedure

In separate procedures, the final preparations were aseptically dispensed in 1 mL aliquots into 5 mL Schott vials (high titre) or 5 mL DIN ampoules (low titre). Stoppers were partially inserted into the containers and the trays of containers loaded onto precooled (-50°C) shelves in a Virtis AdVantage Plus freeze dryer (Biopharma Process Systems) for the high titre preparations or in a CS-100 freeze dryer (Serail) for the low titre ones. The same freeze-drying schedule was used for both freeze-driers. For initial freezing, the products were held at -50°C for 5 h, followed by a further 1 h at -50°C at 0.1 mbar to begin primary drying. The temperature was then ramped up to -20°C over 1 hour and held at -20°C for a further 30 h. Secondary drying proceeded by ramping the temperature up to 25°C over 10 h followed by holding at 25°C for a further 15 h at 0.03 mbar. At the end of freeze drying the containers were backfilled with dry nitrogen gas and the stoppers fully inserted into the containers. For the high titre preparations, vials were removed from the freeze dryer and polypropylene screw caps secured. For the low titre preparations, the ampoules were flame-sealed.

### Nanoparticle tracking system (NTA)

The sucrose-purified supernatant preparation of the LVV_NP-VP35-GP particles was 10-fold serially diluted in PBS to achieve a particle concentration of approximately 10^8^ particles/mL. Reconstituted vials of the freeze/dried preparations LVV_NP-VP35-GP high and LVV_VP40-L high were 10-fold diluted in universal buffer. Samples were analysed using a NanoSight LM10 instrument (Malvern). Samples were injected using a 1mL syringe loaded in the NanoSight syringe pump. Each sample dilution was acquired 5 times for 90-seconds each time and analysed using Nanoparticle Tracking Analysis 2.3 Analytical software. Before each acquisition 100nm polystyrene latex microspheres, diluted in the same buffer of the samples, were used to calibrate the instrument.

### Transmission electron microscopy

55μl of the 1:10 dilution in PBS of the sucrose-purified supernatant containing LVV_NP-VP35-GP particles were loaded into an EM90 rotor (Beckman-Coulter) pre-loaded with freshly plasma-cleaned carbon-coated TEM grids (Agar scientific). The sample was spun at 30 psi (90,000 rpm or 118,000xG _r_max) for 30 minutes in an airfuge (Beckman-Coulter). Grids were removed and washed 3 times for 1 minute in molecular grade water and were stained in 2% aqueous ammonium molybdate for 1 minute. Excess stain was removed and grids were air dried. Images were acquired on a JEM2100 (JEOL Ltd) using a US4000 camera running digital micrograph software (Gatan inc).

### Quantitative reverse-transcriptase polymerase chain reaction

The four freeze-dried HIV-EBOV RNA preparations were reconstituted by adding 1 mL of molecular-grade water. For the in-house assays, high titre preparations were 10-fold serially diluted in universal buffer. The 3^rd^ HIV-1 international standard (NIBSC code 10/152) was reconstituted in 1 mL of molecular water as per the Instructions for Use and 5-fold serially diluted in universal buffer prior to extraction. A 140 μL volume of each sample was extracted using QIAamp viral RNA mini kit (Qiagen) following the manufacturer’s instructions, and eluted in 60 μL AE buffer. Amplification was performed using RNA UltraSense One-Step Quantitative RT-PCR system (Life Technologies), following the manufacturer’s instructions and adding 10 μL of the extracted viral RNA, 0.2 μM of each primer and 0.1 μM of probe (Table 1). Reactions were run on a Mx3005p instrument (Stratagene) for 30 minutes at 50°C, 10 minutes at 95°C, followed by 35 cycles of 30 seconds at 95°C and 90 seconds at 60°C, and analysed using MxPro v.4.1 software. For the commercial assays, 0.85 mL of each sample was extracted using total nucleic acid extraction kit (Roche Diagnostics) in a COBAS® Ampliprep Instrument (Roche Diagnostics), with an elution volume of 75μL. For the Ebola virus *np* target gene, 10μL of each extract was added to 1μL of LIPSGENE ZEBOV kit oligonucleotides (Bioactiva Diagnostica GmbH), 5μL AB TaqMan polymerase (Applied Biosciences) and 4μL molecular grade water in a 96-well micro-titre plate (Roche Diagnostics) and run on a LightCycler 480 II (Roche Diagnostics) for 5 minutes at 50°C, 20 seconds at 95°C, followed by 45 cycles of 95°C for 15 seconds and 60°C for 1 minute. For the Ebola virus *l* gene, 10 μL of the nucleic acid extract was added to 20 μL of the Realstar Filovirus Screen RT-PCR Kit 1.0 (Altona Diagnostics) and run on a Roche LightCycler 480 II for 20 minutes at 50°C, 2 minutes at 95°C, followed by 45 cycles of 95°C for 15 seconds and 60°C for 1 minute. All results were generated on the LightCycler 480 v.1.5.62 software.

**Table 1:** Primers and probes sequences.

Name	Target	Sequence (5’→3’)	Ref.
F565	EBOV NP	TCT GAC ATG GAT TAC CAC AAG ATC	[10]
P567S	EBOV NP	AGG TCT GTC CGT TCA A	[10]
R640	EBOV NP	GGA TGA CTC TTT GCC GAA CAA TC	[10]
EBOV_L_F	EBOV L	AACTGATTTAGAGAAATACAATCTTGC	[18]
EBOV_L_p	EBOV L	ATTGCAACCGTTGCTATGGT	[18]
EBOV_L_R	EBOV L	AATGCATCCAATTAAAAACATTC	[18]
HIV-LTR_F	HIV-1 U5	GCTCTCTGGCTARCTAGGG	
HIV-LTR_p	HIV-1 U5	GCTTCAAGTAGTGTGTGCCC	
HIV-LTR_R	HIV-1 U5	GTTACCAGAGTCACACAACAGA	

Primers and probes used in both quantitative RT-PCR and droplet digital RT-PCR. All probes were labelled 5’-FAM and 3’-BHQ1.

### Droplet digital reverse transcriptase polymerase chain reaction (ddRT-PCR)

Viral RNA extracted for the in-house assays was also analysed by ddRT-PCR. 2 μL of each sample was added to 12.5 μL of One-Step RT-ddPCR kit for probes (Bio-Rad) with 0.9 μM of each primer and 0.125μM of probe (Table 1), 1 μL of 25mM manganese acetate and molecular-grade water to a final volume of 25 μL. To generate the droplets, 20 μL of these solutions was pipetted into a Droplet Generator DG8 Cartridge (Bio-Rad) together with 70 μL of droplet generator oil for probes and loaded in the QX100 Droplet Generator (Bio-Rad). The entire droplet emulsion volume was then loaded in a twin.tec semi-skirted 96-well PCR plate (Eppendorf) and heat sealed with pierceable foil in the PX1™ PCR Plate Sealer and placed in a C1000 Touch™ Thermo Cycler (both from Bio-Rad). Thermal cycling conditions were: 30 minutes at 60°C, 5 minutes at 95°C, followed by 45 cycles of 30 seconds at 94°C and 1 minute at 60°C, and a final step of 10 minutes at 98°C.The droplets were read in a QX100™ droplet reader (Bio-Rad), and analysed using QuantaSoft™ software version 1.7.4.

## Results

### Generation of lentiviral-particles containing Ebola virus RNA

2014 Ebola virus Makona sequences encoding nucleoprotein (NP), viral protein 35 (VP35), glycoprotein (GP), viral protein 40 (VP40) and *l* genes (GenBank KJ660346.2) were *in vitro* synthesised and sub-cloned into a lentiviral vector derived from pSF-lenti (Oxford Genetics) using the restriction enzymes as indicated in Fig 1A. Due to the size limitation of the insert that can be cloned into a lentiviral vector (approximately 9 kilobases), two vectors were produced containing either *np*-*vp35*-*gp* genes or *vp40*-*l* gene sequences. To prevent expression of Ebola virus proteins, each Ebola virus gene lacks its start codon, and contains 3 nucleotide changes which introduce stop codons. Furthermore, the Ebola virus genes have been sub-cloned in the reverse orientation relative to the CMV promoter, driving the expression of the lentiviral genomic RNA. The two lentiviral vectors carrying Ebola virus genes were sequenced using Illumina sequencing technology (GenBank KT186367 for pSF-lenti-NP-VP35-GP and KT186368 for pSF-lenti-VP40-L).

Lentiviral particles containing Ebola virus RNA were produced by transfection of 293T cells with pSF_NP-VP35-GP or pSF_VP40-L together with a packaging plasmid expressing HIV-1 *gag* and *pol* genes. Anti-sense Ebola virus RNA was packaged within the HIV-like particles. To increase the biosafety of the system, the long terminal repeats in the lentiviral vectors are defective (ΔU3), an internal promoter is missing and no envelope protein is expressed in the transfected cells, rendering the HIV-like particles non-infectious.

To confirm that correctly shaped particles were produced with this system, the supernatant containing the HIV-EBOV *np-vp35-gp* RNA particles was analysed using nanoparticle tracking analysis (NTA) (Fig 1B) which determined the average particle size to be 101 nm, similar to previous reports [19] with an estimated concentration of 3.33 x10^10^ particles per millilitre. The same preparation was also visualised by electron microscopy (EM) (Fig 1C) showing spherical particles with evidence of an internal core structure of an average diameter of 116 nm, consistent with that of wild type HIV [20].

### Evaluation of the HIV-EBOV reference materials in NAT assays

Possible plasmid DNA carryover from the transfection was removed from the supernatant by incubation with DNAse I. Particles were then purified by ultracentrifugation on a sucrose cushion and resuspended in the same volume of universal buffer. Universal buffer is a TRIS-HCl solution containing trehalose and human serum albumin; this has been previously used for lyophilised viral NAT reference materials that may be diluted in a number of different clinical matrices [21,22]. Two types of reference materials were produced for each HIV-EBOV RNA preparation: a “high” titre standard, obtained by diluting the viral particle stock 1:10 in Universal buffer and a “low” titre control obtained by diluting the “high” titre preparations 1:10,000 in the same buffer. The four preparations were filled in 1 mL aliquots and freeze-dried following validated standard operating procedures. The appearance of the final products is a white compact cake.

**Fig 2:**
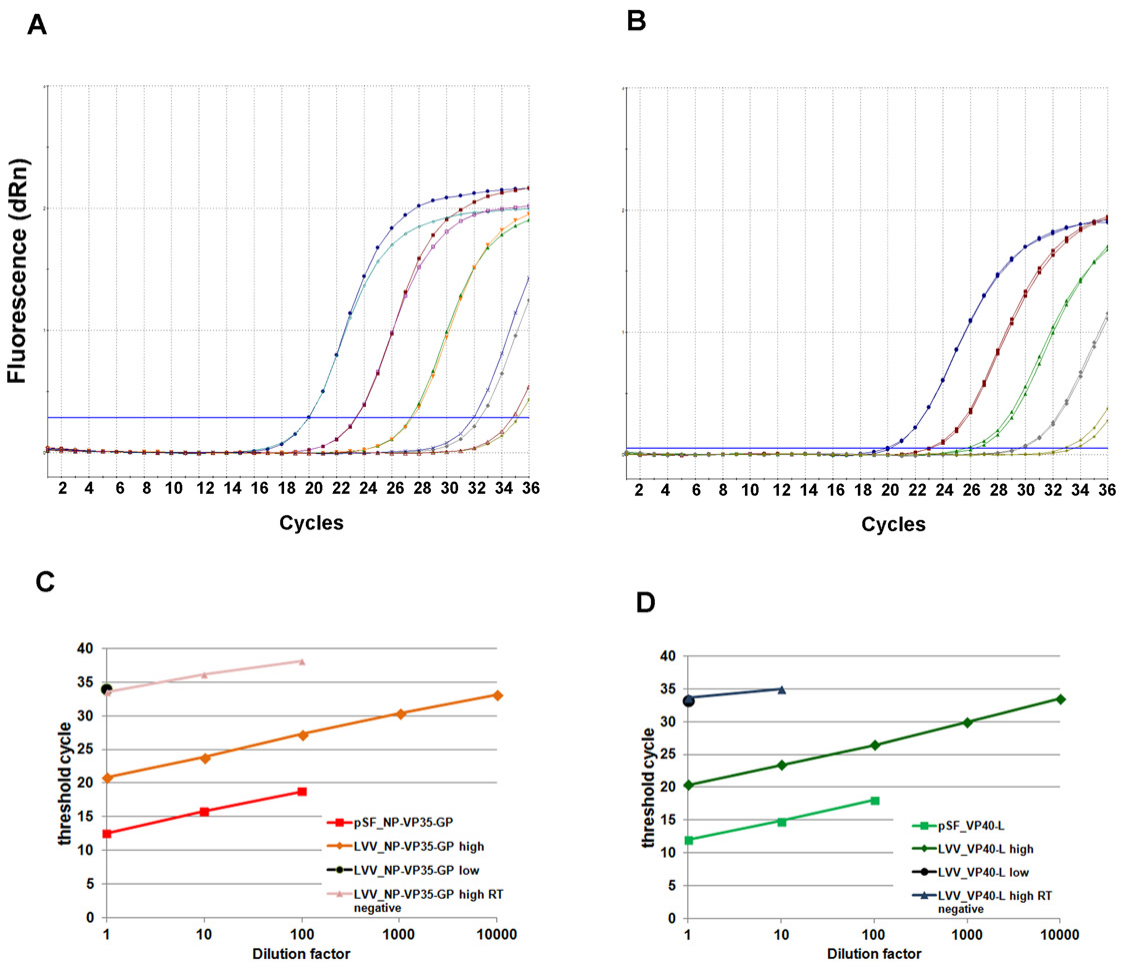
Performance of the HIV-EBOV RNA preparations in qRT-PCR. Representative amplification plot of two 10-fold serially diluted high titre LVV_NP-VP35-GP (A) and LVV_VP40-L (B) vials. Samples from two reconstituted vials per sample were run in duplicate and the mean values of the duplicates plotted as threshold cycle against fluorescence. The dilutions of the high titre LVV_NP-VP35-GP (C) and LVV_VP40-L (D) were also plotted against the dilution factor. Efficiencies of the reaction were calculated based on the slope of the regression line as 102% for *np*-target qRT-PCR and 110% for *I*-target qRT-PCR. The same samples, run without the reverse transcriptase reaction (RT negative), were detectable up to 10^−2^ dilution for LVV_NP-VP35-GP and 10^−1^ for LVV_VP40-L. Three 10-fold dilutions of the plasmids pSF-lenti-NP-VP35-GP and pSF-lenti-VP40-L were run in parallel and the values were the same between RT positive and RT negative qRT-PCR.

The final freeze-dried preparations were evaluated by quantitative RT-PCR (qRT-PCR) using in-house and commercially available assays. In all the assays, two vials of each standard were reconstituted using 1 mL of molecular grade water, and viral RNA was extracted using commercially available kits. In-house assays were developed using published primers and probe sequences for the Ebola virus *np* gene [10] and *l* gene [18]. Ten-fold serial dilutions of the high concentration standards showed good efficiencies and linearity of the qRT-PCR for both targets (Fig 2A and 2B). In parallel, the same preparations were evaluated using commercial assays: RealStar® filovirus RT-PCR kit (Altona Diagnostics)-authorised by the Food and Drug administration for emergency use [23], and targeting Ebola virus *l* gene, and LIPSGENE ZEBOV kit (Bioactiva Diagnostica GmbH) targeting the *np* gene. Each preparation produced the expected results using both in-house and commercial assay (Table 2): the difference in the threshold cycle (Ct) between the high titre and the low titre was 13.2 cycles for the lentiviral particles carrying Ebola virus *np-vp35-gp* (LVV_NP-VP35-GP) and 13.0 cycles for *vp40-l* genes (LVV_VP40-L), using the in-house assays. Assuming an efficiency of reaction of 100%, this corresponds to 10,000-fold difference in the target concentration between the high and low titre preparations. Furthermore, no cross contamination (Ct >35) was observed when the LVV_NP-VP35-GP materials were assessed in the *l*-based assays. Conversely, no *l* sequences were detected in the *np*-based assays for the LVV_VP40-L materials (Table 2).

**Table 2:** Evaluation of the HIV-EBOV RNA standard by quantitative RT-PCR.

	in-house		Lipsgene	RealStar
Target	*np*	*l*	*np*	*l*
**LVV_NP-VP35-GP high**	19.9±0.2	>35	23.0±0.7	>35
**LVV_NP-VP35-GP low**	33.1±0.3	>35	34.5±0.1	>35
**LVV_VP40-L high**	>35	20.5±0.2	>35	20.4±0.5
**LVV_VP40-L low**	>35	33.6±0.5	>35	33.2±0.02

Viral RNA samples from three independent extractions were run in duplicate and the results expressed as average of the threshold cycle (Ct) ± standard deviation.

In order to assess any plasmid DNA carryover contaminations, reactions were performed without the reverse transcriptase step (RT-negative). A DNA signal was detected in the high titre preparations, and was about 10,000 times lower than the corresponding RNA signal (Fig 2C and 2D). The presence of DNA in the high titre reference material despite DNase I treatment may be attributable to the small amount of viral DNA contained within a lentiviral particle [24–26].

### Relative quantification of the HIV-EBOV RNA reference materials

The subcloning of the Ebola virus target genes across two separate reference preparations could lead to incongruence in the interpretation of the results, due to differences in Ebola virus gene-specific PCR efficiencies. To estimate the equivalence of Ebola virus sequences across the reference preparations, we took advantage of the WHO 3^rd^ HIV-1 International Standard (NIBSC 10/152) which shares the HIV-1 long terminal repeat (LTR) sequences present within the HIV-EBOV RNA reference materials. Using HIV-1 LTR specific primers and probe targeting the common sequences, the 3^rd^ HIV-1 International Standard was used as a reference for estimating the equivalence of sequences across the HIV-EBOV RNA reference preparations.

**Fig 3:**
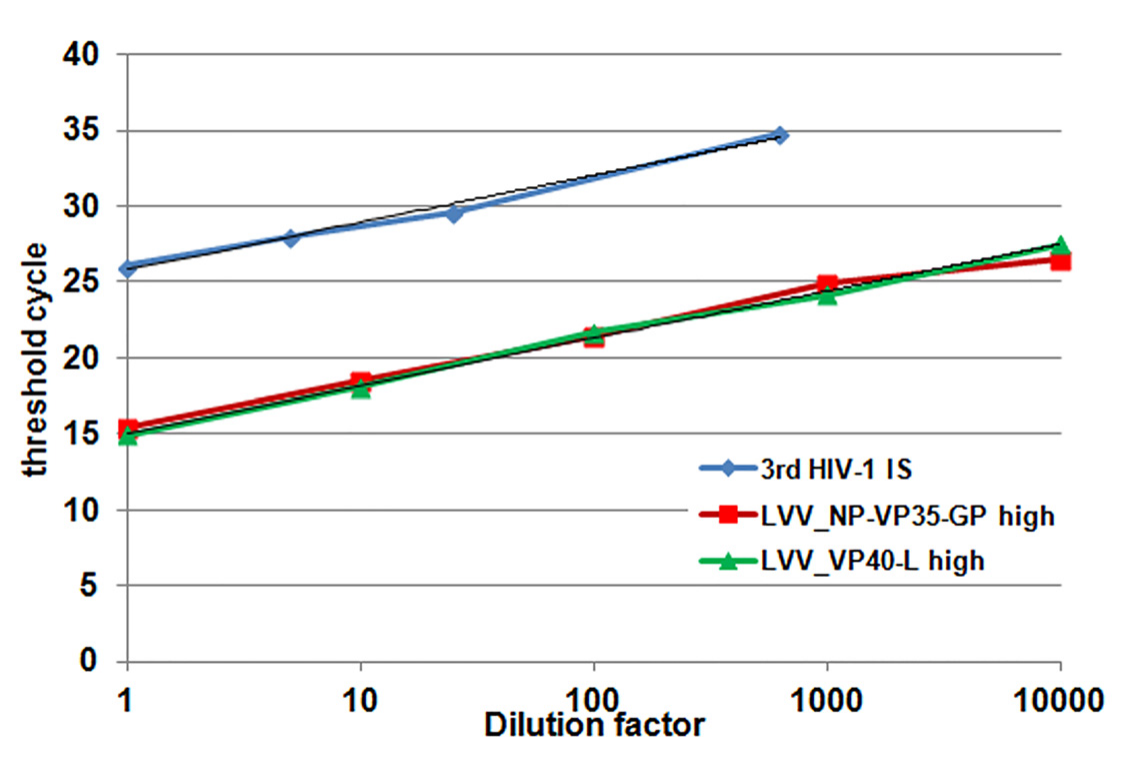
Efficiency of the quantitative RT-PCR targeting HIV LTR. Viral RNA extracted from serial dilutions of WHO 3^rd^ HIV-1 International standard (dilution factor 5, blue), LVV_NP-VP35-GP high (dilution factor 10, red) and LVV-VP40-L (dilution factor 10, green) were processed in duplicate in a quantitative RT-PCR using primers and probes annealing within the U5 region of the HIV-1 LTR. The graph shows a representative result of 3 independent experiments; the efficiencies of the reaction between samples were similar as represented by the slope of linear regression: HIV-1 IS = 3.122, LVV_NP-VP35-GP = 3.142, LVV_VP40-L = 3.110.

Prior to viral RNA extraction, freshly reconstituted vials of the HIV-EBOV RNA reference materials and the 3^rd^ HIV-1 International Standard were serially diluted in universal buffer. The viral RNA was processed in a qRT-PCR, and the efficiencies of the reactions were found to be similar (Fig 3). When expressed relative to the 3^rd^ HIV-1 International Standard (Table 3), the ratio between HIV-EBOV RNA high titre preparations LVV_VP40-L and LVV_NP-VP35-GP was 2.3, while for the low titre preparations the ratio was 2.94. Similar to the results obtained using the Ebola virus specific qRT-PCR (Table 2), the high low titre reference materials differed in concentration by approximately 10,000-fold.

**Table 3:** Examples of the HIV-EBOV RNA standard values using different technologies.

	qRT-PCR HIV LTR	ddRT-PCR HIV LTR	qRT-PCR *np/l*	ddRT-PCR *np*	NTA
	International Unit/mL	copies/mL	copies/mL	copies/mL	particles/mL
**LVV_NP-VP35-GP high**	1.92±0.35 x 10^8^	0.87±0.04 x 10^8^	4.40±0.19 x 10^8^	0.64±0.02 x 10^8^	4.24±0.10 x 10^9^
**LVV_NP-VP35-GP low**	1.90±0.68 x 10^4^	0.98±0.03 x 10^4^	4.09±0.31 x 10^4^	0.68±0.11 x 10^4^	n/d
**LVV_VP40-L high**	4.39±0.35 x 10^8^	1.5±0.45 x 10^8^	3.39±0.15 x 10^8^	n/d	3.63±0.11 x 10^9^
**LVV_VP40-L low**	5.64±0.78 x 10^4^	2.88±1.24 x 10^4^	4.67±0.41 x 10^4^	n/d	n/d

Quantitative RT-PCR (qRT-PCR) was performed in duplicate in three independent experiments using HIV-1 LTR specific primers and probe; samples were quantified against the 3^rd^ HIV-1 International standard (assigned value 185 000 IU/mL) run in parallel. The same samples and set of primers and probe were used in a droplet digital RT-PCR (ddRT-PCR) and results expressed as average of two independent experiments run in duplicate. HIV-EBOV RNA preparations were also quantified using Ebola virus *np* or *I* specific primers and probes (qRT-PCR np/l) and copies per mL were inferred using standard curves obtained by serial dilution of the lentiviral plasmids used to produce the particles. Ebola virus *np*-specific primers and probe were also used in a ddRT-PCR. Both HIV-EBOV RNA high titre preparations were also analysed by NTA. Results are reported as average concentration±standard deviation calculated on 5 acquisitions of a 1:10 dilution in universal buffer. Where results are reported as ‘copies/mL’, the relationship to genuine genome equivalence numbers is unknown.

HIV-EBOV RNA preparations were also analysed using another NAT-based assay, droplet digital RT-PCR (ddRT-PCR) using the same sets of primers and probe employed for the qRT-PCR; this method is based on Poisson distribution and can provide an absolute copy number in absence of a calibrator. Each sample was analysed using two sets of primers and probe, one targeting the HIV-1 LTR and one for the Ebola virus gene *np*. Serial dilutions of the high titre HIV-EBOV RNA standard confirmed the linearity of the assay for both set of primers and probe (data not shown). Ebola virus *l* gene-specific primers and probe used in the qRT-PCR did not perform efficiently in the ddRT-PCR, possibly due to a lower than recommended melting temperature. Optimisation of the RT-PCR conditions could overcome this problem, however this is beyond the scope of this work.

In Table 3 are summarised the values obtained for the HIV-EBOV RNA preparations in the different assays, expressed in the readout unitage of each assay. Where results are reported as ‘copies/mL’, the relationship to genuine genome equivalence numbers is unknown. Copies reported in one assay are not necessarily equivalent to copies reported in another. Furthermore, there is no conversion factor between International Units/mL or copies/mL. In all cases the ratio between the high and low titre preparations was about 10,000-fold, as expected. All the samples were detectable using both qRT-PCR and ddRT-PCR technologies using different set of primers and probe with results in the same order of magnitude. The high concentration preparations were also analysed by NTA in universal buffer. The particle concentrations were about 10 times higher than values estimated with molecular methods (Table 3) suggesting that about 10% of the particles contained HIV-EBOV viral genome.

### Stability study of the HIV-EBOV RNA reference materials

Stability of the HIV-EBOV RNA reference materials at different temperatures was investigated to simulate shipping conditions. Three vials for each preparation were stored at different temperatures from -70°C to 56°C and tested after two weeks in parallel using in-house qRT-PCR for the *np* and *l* genes. Each freeze-dried preparation was easily reconstituted. The results are reported as average of the difference in the threshold cycle (ΔCt) in comparison to the values obtained when the samples were stored at -70°C (Table 4). Student t test was performed on each set of data and both high titre standards at 45°C and 56°C and the LVV_VP40-L low titre at 56°C were found to differ significantly from the baseline values. Results obtained from this initial stability study indicated that the reference materials were suitably stable for storage at -20°C and short-term shipment at ambient temperatures up to 37°C. It is noteworthy that the low titre preparations were more stable at higher temperatures (Table 4). For future development, we aim to optimise the filling and freeze-drying conditions in order to further improve the stability of the HIV-EBOV RNA reference preparations.

**Table 4:** Stability of the freeze-dried preparations after two weeks of storage at different temperatures.

	-70°C	-20°C	4°C	20°C	37°C	45°C	56°C
**NP-VP35-GP high**	0	-0.13	-0.08	-0.17	0.23	**1.14**	**7.40**
**NP-VP35-GP low**	0	-0.17	-0.19	-0.36	-0.61	-0.36	0.02
**VP40-L high**	0	-0.16	-0.21	-0.19	0.25	**1.77**	**9.10**
**VP40-L low**	0	0.01	-0.19	-0.24	0.03	0.29	**0.75**

Samples were assessed by quantitative RT-PCR against *np* or *l* gene and ΔCt are calculated as the difference: Ct (°T)-Ct (-70°C). The data in the table represent the average of three independent experiments. In bold the ΔCt values which were significant by Student t test (p<0.001).

## Discussion

NAT-based diagnostic techniques have a crucial role during the on-going Ebola virus outbreak in Western Africa [2,3]. Reference materials are needed to assess the validity of the assays used, to compare results across assays and to provide guidance to the regulatory agencies in the evaluation of new assays. It is crucially important that Ebola virus NAT reference materials standardise and control the entire process from the extraction to the final amplification and detection reaction.

In this study we report the development of safe, non-infectious, stable reference materials for Ebola virus NAT-based assays. This has been achieved by incorporating Ebola virus RNA into HIV-1 like particles. These chimaeric particles, resembling spherical HIV-1 particles on the outside, have an internal core containing Ebola virus genes as anti- sense RNA within the two HIV-1 LTRs. The lack of any viral Envelope protein and HIV-1 structural genes renders this material non-infectious and unable to replicate. To further increase the safety of these preparations, the cloned Ebola virus gene sequences were designed to lack the start codon and contained random stop codons, ensuring that full length Ebola virus protein could not be produced. The plasmids used to generate the particles were fully sequenced and the data are available in the GenBank database to allow end users to check the suitability of the reference material for their assay.

Four freeze-dried reference materials were produced: two high-titre materials containing either *np-vp35-gp* (LVV_NP-VP35-GP) or *vp40-l* (LVV_VP40-L) sequences, and two corresponding low-titre materials representing 10,000-fold dilutions of the high titre materials. The high-titre materials would be suitable as a standard for the characterisation and calibration of diagnostic NAT assays, while the low-titre preparations were designed to serve as external in-run controls. When tested in quantitative RT-PCR assays, the reference materials were shown to be suitable for purpose. Cross-contamination between the two types of preparations was not observed. DNA contamination was detectable at a dilution of 1:10,000. Similar results were obtained with the 3^rd^ HIV-1 International Standard (data not shown). Presence of DNA within lentiviral particles is known to occur in this system [24–26], although its contribution to the signal intensity is negligible compared to that of the RNA.

The freeze-dried HIV-EBOV RNA reference materials were stable at temperatures up to 37°C for 2 weeks, making them suitable for shipping at ambient temperature; however temperature control during shipment to hot countries may still be required, since at 45°C a loss of activity after 2 weeks was observed for the high titre standards. Similar studies have demonstrated that lentiviral freeze-dried preparations can be stored up to 20°C for up to 6 months without significant loss of signal [27,28], making these reference materials ideal in regions where continuity of the cold-chain cannot always been guaranteed. The stability assessment is on-going to determine the real-time stability of the Ebola virus reference materials.

Our study indicates that the Ebola virus reference materials are suitable for inclusion in an International collaborative study currently being organised by NIBSC on behalf of the WHO. The aim of the WHO study is to assess the suitability of different Ebola virus RNA preparations to serve as International Standards, with an assigned concentration in International Units (IU) per mL, for use in the harmonisation of Ebola virus NAT assays. To this end, our results support the use of the 3^rd^ HIV-1 International Standard as a reference to nominally assign to the Ebola virus reference materials unitage in arbitrary terms of the IU. The WHO study will also involve the characterisation of the standard preparations in terms of reactivity and specificity in different assay systems and assessment of commutability; this will establish the extent to which each preparation is suitable to serve as an international standard for the variety of different samples and assay types.

A potential limitation of the HIV-EBOV RNA reference materials described in this work is that not every gene in the Ebola virus genome is included in the preparations. Ebola virus genes *np*, *vp35*, *gp*, *vp40* and *l* were chosen based on information on current assay targets; however, since for commercial diagnostic kits the target gene is not specified, the production of additional versions of HIV-EBOV RNA particles incorporating other target Ebola virus genes may be required. However, one of the main advantages of this system is its flexibility: as soon as the target sequence is available, the reference material can be produced using the same approach in a relatively short amount of time. Furthermore, the presence of residual HIV derived sequences in the vector allows the relative potency of each Ebola virus vector to be determined and ensures that reference materials can be produced in equimolar ratios. This feature will enable the relative sensitivity of diagnostic assays targeting any region of the Ebola virus genome to be established.

The lentiviral packaging system represents a safe, stable and rapid tool to create reference materials for highly pathogenic RNA viruses which can also be employed as part of preparedness plans for future outbreaks.
